# Supplementary material for: Therapeutic Options and Outcomes for the Treatment of Children with Gram-Positive Bacteria with Resistances of Concern: A Systematic Review
Source: Antibiotics (Basel). 2023 Jan 28;12(2):261. doi: 10.3390/antibiotics12020261 (PMC9952189; doi:10.3390/antibiotics12020261)
Supplement: Supplementary file 1 [file antibiotics-12-00261-s001.zip › PICO Gram positive additional file S2.pdf]

PICO

Participants/population
Children, term and preterm newborns with confirmed Gram-positive multidrug resistant organisms (GP-MDROs) infections that were receiving antimicrobial treatment and presenting clinical and/or microbiological outcomes clearly specified.

Intervention(s)/Exposure(s)
Any antimicrobial treatment clearly defined

Comparator(s)/Control
Standard of care at the time and place where the study was done
Not applicable

Types of study to be included
Randomized control trials, control and non-control before and after studies, control and non-control interrupted time series, case series, case reports and cohort studies will be included for review.
Exclusion: Review, Notes and letters, Conference abstracts, Opinion articles, Studies from which it is not possible to extract neonatal data, studies about MDR on malaria, HIV, tuberculosis viral and fungal treatment.

Main outcome
The primary outcome is the mortality related GP-MDROs infection.

Additional outcomes
The secondary outcomes are clinical success, defined as complete resolution or substantial improvement of the signs and symptoms of the index infection, microbiological success measured by suppression, eradication or relapses of bacterial growth, and treatment-related adverse effects
