# Supplementary material for: Therapeutic Options and Outcomes for the Treatment of Children with Gram-Positive Bacteria with Resistances of Concern: A Systematic Review
Source: Antibiotics (Basel). 2023 Jan 28;12(2):261. doi: 10.3390/antibiotics12020261 (PMC9952189; doi:10.3390/antibiotics12020261)
Supplement: Supplementary file 1 [file antibiotics-12-00261-s001.zip › search strategy additional file S1.pdf]

1. (newborn* or neonat* or infan* or toddler* or pre-schooler* or preschooler* or child* OR children or adolescen* or pediatr* or paediatr* or youth* or teen or teens or teenage* or kid or kids or baby or babies).mp. [mp=title, abstract, original title, name of substance word, subject heading word, floating sub-heading word, keyword heading word, protocol supplementary concept word, rare disease supplementary concept word, unique identifier, synonyms]
2. (anti-bacterial* or antibacterial*or antibiotic* or anti-infective or bactericid* or bacteriocid* or antimicrobial* or treatment* or therap* or prophyla* or perioperative*).mp. [mp=title, abstract, original title, name of substance word, subject heading word, floating sub-heading word, keyword heading word, protocol supplementary concept word, rare disease supplementary concept word, unique identifier, synonyms]
3. (vancomycin or teicoplanin or oxacillin or cloxacillin or dicloxacillin or flucloxacillin or nafcillin or cefazolin or clindamycin or quinupristin dalfopristin or quinupristin-dalfopristin linezolid or trimethoprim? sulphamethoxazole or trimethoprim sulphamethoxazole or daptomycin or tedizolid or dalbavancin or clindamycin? or methicillin? oritavancin).mp
4. (amoxicillin? or amoxicillin-clavulanate combination? Or ampicillin? Or ampicillin-sulbactam or beta lactams or clavulanic acid? Piperacillin or piperacillin-tazobactam Or cefaclor? or cefadroxil? or cefamandole? or cefatrizine? or cefazolin? or cefixime? or cefmenoxime? or cefmetazole? or cefonicid? or cefoperazone? or cefotaxime? or cefotetan? or cefotiam? or cefoxitin? or cefsulodin? or ceftazidime? or ceftriaxone? or cefuroxime? or cephacetrile? or cephalexin? or cephaloglycin? or cephaloridine? or cephalosporin? or cephalothin? or cephamycin? or cephapirin? or ceftalozane tazobactam or Ceftazidime Avibactam or ceftaroline or ceftobiprole or chloramphenicol? or clarithromycin? azithromycin? Imipenem cilastatin relebactam or aztreonam? or bacitracin? or cloxacillin? or colistin? or dactinomycin? or doxycycline? or erythromycin? or floxacillin? or fluoroquinolone? norfloxacin? Or ciprofloxacin or levofloxacin or fosfomycin? or fusidic acid? or gentamicin? or imipenem? Or meropenem or ertapenem or meropenem-vaborbactam or josamycin? or lincomycin? or lincosamide? or mupirocin? or nafcillin? or netilmicin? or ofloxacin? or paromomycin? or penicillin? or piperacillin? or polymyxin? or streptomycin? or sulbactam? Or tetracycline? or thiamphenicol? or ticarcillin? or tobramycin? or tigecycline or colistin)
5. exp Gram-negative Bacterial Infection/ or gram negative bacterial infection*.mp.
6. exp Enterobacteriaceae/ or enterobacteriaceae.mp.
7. exp Brucella/ or brucella.mp.
8. exp Fusobacterium/ or fusobacteriaceae.mp
9. exp Peptostreptococcus/ or peptostreptococcus.mp
10. exp Enterobacter aerogenes/ or enterobacter.mp. or exp Enterobacter/ or exp Enterobacter cloacae/
11. exp Bacillus/ or bacillus. Mp. or exp bacillus anthracis/
12. exp Escherichia/ or escherichia.mp. or exp Escherichia coli/
13. klebsiella.mp. or exp Klebsiella/ or exp Klebsiella pneumoniae/ or exp Klebsiella oxytoca/
14. Haemophilus.mp. or exp haemophilus/ or exp haemophilus influenzae/ or exp Haemophilus ducreyi/
15. listeria.mp. or exp listeria/ or exp Listeria monocytogenes/

16. clostridium.mp. or exp clostridium difficile/ or exp Clostridium perfringens/ or exp Clostridium botulinum/ or exp Clostridium tetani/
17. exp Fusobacterium/ or fusobacterium.mp.
18. exp Peptostreptococcus/ or peptostreptococcus.mp.
19. exp Bacillus/ or bacillus.mp. or exp bacillus anthracis/
20. exp Morganella morganii/ or exp Morganella/ or morganella.mp.
21. proteus.mp. or exp Proteus/
22. serratia.mp. or exp Serratia/ or exp Serratia marcescens/
23. acinetobacter.mp. or exp Acinetobacter baumannii/ or exp Acinetobacter/
24. citrobacter.mp. or exp Citrobacter freundii/ or exp Citrobacter/ or exp Citrobacter rodentium/ or exp Citrobacter koseri/
25. exp Pseudomonas aeruginosa/ or exp Pseudomonas/ or pseudomonas.mp
26. exp Gram-positive Bacterial Infection/ or gram positive bacterial infection*.mp.
27. Streptococci.mp. or exp Streptococcus viridans/ or exp Streptococcus pneumoniae/ or exp streptococcus agalactiae/ or exp streptococcus pyogenes/
28. staphylococcus.mp. or exp staphylococcus aureus/ or exp staphylococcus epidermidis/ or exp staphylococcus capitis/ or exp staphylococcus haemolyticus/ or exp staphylococcus saprophyticus/ or exp staphylococcus lugdunensis/ or exp staphylococcus hominis/ or exp staphylococcus hyicus/
29. Enterococcus.mp. or exp Enterococcus faecalis/ or exp enterococcus faecium/ or exp enterococcus hirae/
30. Nocardia. mp. Or exp nocardia/
31. rhodococcus.mp. or exp rhodococcus/
32. actinomyces. Mp. Or exp actinomyces/
33. 5 or 6 or 7 or 8 or 9 or 10 or 11 or 12 or 13 or 14 or 15 or 16 or 17 or 18 or 19 or 20 or 21 or 22 or 23 or 24 or 25 or 26 or 27 or 28 or 29 or 30 or 31 or 32
34. extended spectrum beta-lactamases.mp.
35. ESBL.mp.
36. exp beta-Lactamases/ or carbapenemase.mp.
37. carbapenem resistance.mp.
38. carbapenem resistant.mp.
39. drug resistance.mp. or exp Drug Resistance/
40. carbapenemase*.mp. [mp=title, abstract, original title, name of substance word, subject heading word, keyword heading word, protocol supplementary concept word, rare disease supplementary concept word, unique identifier, synonyms]
41. (carbapenem adj1 resist*).mp. [mp=title, abstract, original title, name of substance word, subject heading word, keyword heading word, protocol supplementary concept word, rare disease supplementary concept word, unique identifier, synonyms]
42. CTX-M.mp.
43. AmpC.mp.
44. MBL.mp.
45. metallo-b-lactamase.mp.
46. vim.mp.

47. NDM.mp.
48. OXA.mp.
49. IMP.mp.
50. KPC.mp.
51. Klebsiella pneumoniae carbapenemase.mp.
52. TEM.mp.
53. SHV.mp.
54. methicillin resistant.mp.
55. Methicillin Resistance.mp
56. MRSA.mp.
57. MSSA.mp.
58. Vancomycin resistance. mp
59. 34 or 35 or 36 or 37 or 38 or 39 or 40 or 41 or 42 or 43 or 44 or 45 or 46 or 47 or 48 or 49 or 50 or 51 or 52 or 53 or 54 or 55 or 56 or 57
60. 1 AND 2 AND 33 AND 59
61. exp "Outcome Assessment (Health Care)"/ or outcome*.mp. [mp=title, abstract, original title, name of substance word, subject heading word, floating sub-heading word, keyword heading word, protocol supplementary concept word, rare disease supplementary concept word, unique identifier, synonyms]
62. exp Mortality/ or mortality.fs. or (mortalit* or death* or fatal*).mp. or Morbidity/ or morbidit*.mp
63. exp "Drug-Related Side Effects and Adverse Reactions"/ or adverse effects.fs. or (adverse effect* or adverse reaction* or adverse drug reaction* or adverse event* or adverse drug event* or undesirable effect* or side effect*).ti,ab,kw.
64. microbiological eradication/ or microbiolog* eradication.mp.
65. microbiological failure/or microbiolog* failure.mp.
66. microbiological relapse/or microbiolog* relapse.mp.
67. 61 or 62 or 63 or 64 or 65 or 66
68. 60 AND 67
69. 68 not animals.mp. [mp=title, abstract, original title, name of substance word, subject heading word, keyword heading word, protocol supplementary concept word, rare disease supplementary concept word, unique identifier, synonyms]
